# Assessment of clinical effect and treatment quality of immediate-release carvedilol-IR versus SLOW release carvedilol-SR in Heart Failure patients (SLOW-HF): study protocol for a randomized controlled trial

**DOI:** 10.1186/s13063-018-2470-5

**Published:** 2018-02-13

**Authors:** Dong-Ju Choi, Chan Soon Park, Jin Joo Park, Hae-Young Lee, Seok-Min Kang, Byung-Su Yoo, Eun-Seok Jeon, Seok Keun Hong, Joon-Han Shin, Myung-A Kim, Dae-Gyun Park, Eung-Ju Kim, Soon-Jun Hong, Seok Yeon Kim, Jae-Joong Kim

**Affiliations:** 1 Department of Internal Medicine, Seoul National University College of Medicine, Seoul National University Bundang Hospital, Seongnam, South Korea; 2 Department of Internal Medicine, Seoul National University College of Medicine, Seoul National University Hospital, Seoul, South Korea; 3 Division of Cardiology, Yonsei University Severance Hospital, Seoul, South Korea; 4 Division of Cardiology, Yonsei University Wonju Severance Christian Hospital, Wonju, South Korea; 5 Department of Internal Medicine, Sungkyunkwan University College of Medicine, Samsung Medical Center, Seoul, South Korea; 6 Division or Cardiology, Sejong General Hospital, Bucheon, Gyeonggi-do South Korea; 7 Division of Cardiology, Ajou University Hospital, Suwon, Gyeonggi-do South Korea; 8 Department of Internal Medicine, Seoul National University College of Medicine, Seoul National University Boramae Medical Center, Seoul, South Korea; 9 Cardiovascular Center, Hallym University Medical Center, Seoul, South Korea; 10 Division of Cardiology, Korea University Guro Hospital, Seoul, South Korea; 11 Division of Cardiology, Korea University Anam Hospital, Seoul, South Korea; 12 Department of Internal Medicine, Seoul Medical center, Seoul, South Korea; 13 Department of Internal Medicine, University of Ulsan College of Medicine, Asan Medical Center, Seoul, South Korea; 14 Cardiovascular Center and Division of Cardiology, Department of Internal Medicine, Seoul National University Bundang Hospital, 82 Gumi-ro 173 Beon-gil, Bundang-gu, Gyeonggi-do 13620 South Korea

**Keywords:** Heart failure with reduced ejection fraction, Carvedilol, Slow release, Immediate release, Clinical efficacy, NT-proBNP

## Abstract

**Background:**

Carvedilol is a non-selective, third-generation beta-blocker and is one of the cornerstones for treatment for patients with heart failure and reduced ejection fraction (HFrEF). However, due to its short half-life, immediate-release carvedilol (IR) needs to be prescribed twice a day. Recently, slow-release carvedilol (SR) has been developed. The aim of this study is to evaluate whether carvedilol-SR is non-inferior to standard carvedilol-IR in terms of its clinical efficacy in patients with HFrEF.

**Methods/design:**

Patients with stable HFrEF will be randomly assigned in a 1:1 ratio to the carvedilol-SR group (160 patients) and the carvedilol-IR group (160 patients). Patients aged ≥ 20 years, with a left ventricular ejection fraction ≤ 40%, N-terminal pro B-natriuretic peptide (NT-proBNP) ≥ 125 pg/ml or BNP ≥ 35 pg/ml, who are clinically stable and have no evidence of congestion or volume retention, will be eligible. After randomization, patients will be followed up for 6 months. The primary endpoint is the change in NT-proBNP level from baseline to the study end. The secondary endpoints include the proportion of patients with NT-proBNP increment > 10% from baseline, composite of all-cause mortality and readmission, mortality rate, readmission rate, changes in blood pressure, quality of life, and drug compliance.

**Discussions:**

The SLOW-HF trial is a prospective, randomized, open-label, phase-IV, multicenter study to evaluate the therapeutic efficacy of carvedilol-SR compared to carvedilol-IR in HFrEF patients. If carvedilol-SR proves to be non-inferior to carvedilol-IR, a once-daily prescription of carvedilol may be recommended for patients with HFrEF.

**Trial registration:**

ClinicalTrials.gov, ID: NCT03209180. Registered on 6 July 2017.

**Electronic supplementary material:**

The online version of this article (10.1186/s13063-018-2470-5) contains supplementary material, which is available to authorized users.

## Background

Heart failure (HF) is a clinical syndrome characterized by typical symptoms and signs that result from abnormal cardiac structure or function [1]. It is globally present with an increasing prevalence and is associated with high morbidity and mortality [2, 3]. The current treatment of HF targets includes the prevention of acute decompensation and improving the long-term survival of HF patients [4].

Neuro-humoral activation plays a crucial role in development and progression of HF [5]. Beta-blockers reduce the increased sympathetic tone in HF patients and have been shown to improve the survival in patients with HF and a reduced ejection fraction (HFrEF) [6–8]. Carvedilol is a non-selective beta-blocker which has been most extensively studied in HF patients with mild-to-severe left ventricular (LV) dysfunction [8, 9]. Carvedilol has also shown additional mortality risk reduction in HFrEF patients compared to metoprolol [10].

Current HF guidelines recommend the administration of carvedilol twice daily [1, 11]. This is due to rapid absorption and metabolism of carvedilol whose plasma concentration reaches a peak within 1–2 h after administration and shows an elimination half-life of 7–10 h after peak concentration [12]. Although the drug compliance of carvedilol was comparable between once- and twice-daily dosing in one report [13]; however, there has been a concern that regimen complexity is inversely associated with medication adherence and related to even worse clinical outcomes in other studies and real-world clinical practice [14–16].

In the “Assessment of clinical effect and treatment quality of immediate-release carvedilol-IR versus SLOW-release carvedilol-SR in Heart Failure patients (SLOW-HF): a prospective, randomized, open-label, multicenter study,” we aim to evaluate the clinical efficacies of immediate-release carvedilol (IR) and slow-release carvedilol (SR) forms of carvedilol in Asian HFrEF patients.

## Methods/design

### Overview

**Fig. 1:**
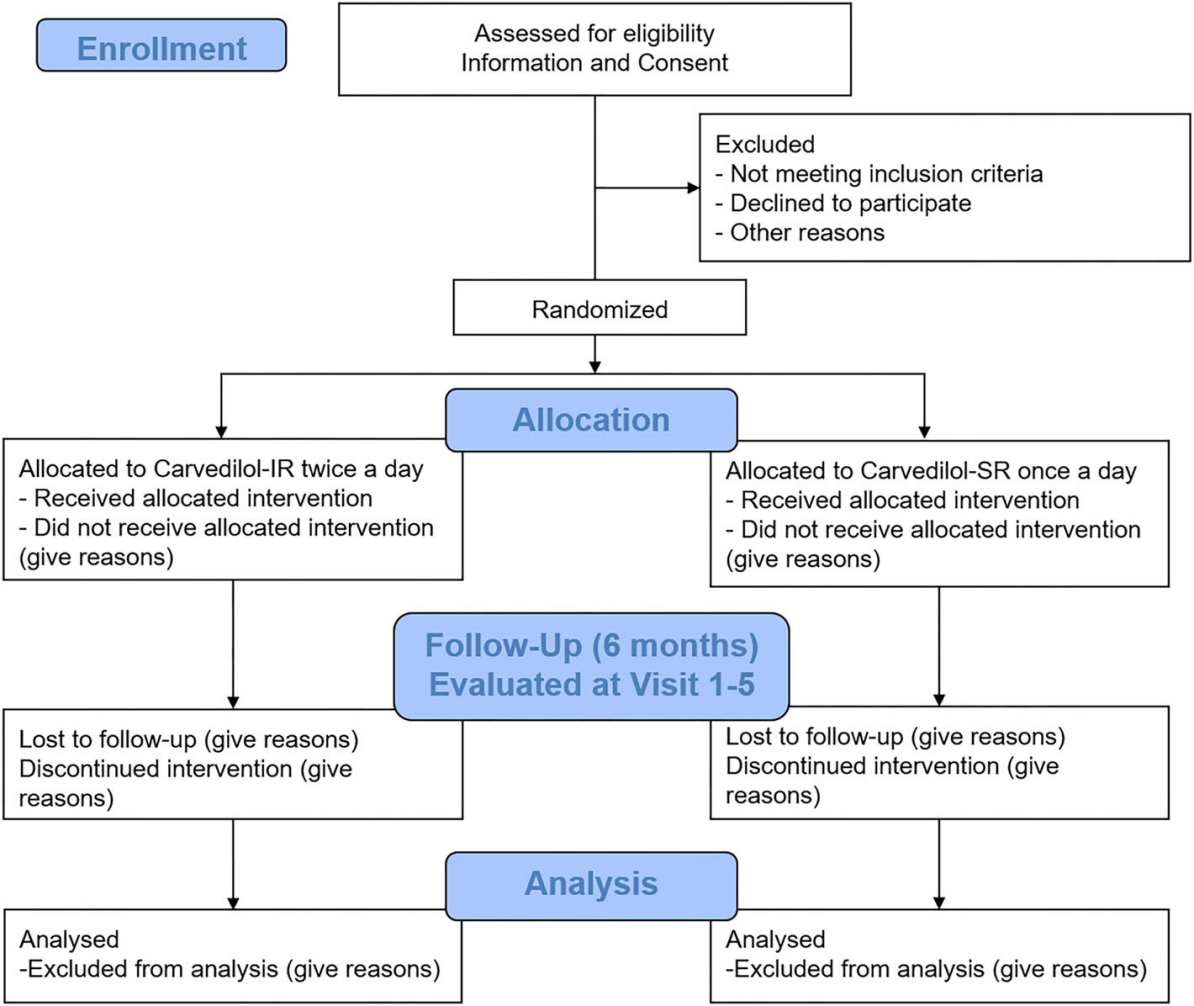
Study flow chart. *BNP* B-type natriuretic peptide, *HFrEF* heart failure with reduced ejection fraction, *IR* immediate-release, *LVEF* left ventricular ejection fraction, *NT-proBNP* N-terminal B-type natriuretic peptide, *SR* slow-release

The study flow is presented in Fig. 1. This trial is a prospective, randomized, open-label, phase-IV, multicenter trial to evaluate the efficacy of carvedilol-SR. The aim of this study is to evaluate whether carvedilol-SR is non-inferior to standard carvedilol-IR in terms of its clinical efficacy in patients with HFrEF. The protocol of this trial has been registered at ClinicalTrials.gov (registration number: NCT03209180). The protocol follows the Standard Protocol Items: Recommendations for Interventional Trials (SPIRIT) Checklist (Additional file 1).

### Study population

Patients who are at aged 20 years or older and with a confirmed diagnosis of HFrEF (left ventricular ejection fraction (LVEF) ≤ 40%) within the pre-analytical 6 months will be included in this study. Transthoracic echocardiography was operated at each institute according to the recommendations of the American Society of Echocardiography using commercially available systems [17]. In addition, the patients should be clinically stable without evidence of congestion and fluid retention, and have an increased level of N-terminal B-type natriuretic peptide (NT-proBNP) or BNP. Blood sampling and tests including NT-proBNP were conducted by laboratories at each participating institute which were certified by The Korean Association of Quality Assurance for Clinical Laboratory. After enrollment, measurement of NT-proBNP will be performed with an electro-chemiluminescence immunoassay method using cobas® 8000 (Roche Diagnostics, Mannheim, Germany) in a central laboratory. Patients will be enrolled at 13 tertiary referral centers in the Republic of Korea. Patients will be excluded if they have any of followings: low blood pressure (sitting systolic blood pressure < 90 mmHg), bradycardia (resting heart rate < 50 beats/min), ischemic heart disease (unstable angina, myocardial infarction) within 1 month, severe cerebrovascular accident, respiratory diseases with bronchospasm, and peripheral vascular diseases. The detailed inclusion and exclusion criteria are listed in Table 1.

**Table 1:** Inclusion and exclusion criteria

Inclusion criteria	
1	Men or women aged 20 years or older
2	Confirmed left ventricular ejection fraction ≤ 40% by echocardiography within the pre-analytical 6 months
3	NT-proBNP level ≥ 125 pg/ml or BNP level ≥ 35 pg/ml within the pre-analytical 3 months
4	Clinically stable patient without evidence of congestion or extracellular fluid retention
5	Patients providing written informed consent
Exclusion criteria	
1	Systolic blood pressure in a sitting position < 90 mmHg or resting heart rate < 50 beats/min at screening
2	Patient has a contraindication to beta-blockers
3	Patient who are expected to take another beta-blocker after randomization
4	Cardiovascular diseases; Ischemic heart disease (unstable angina, myocardial infarction) within 1 month Hypertrophic cardiomyopathy Cor pulmonale Hemodynamically significant stenosis of the aorta, aortic valve, or mitral valve Acute myocardial infarction with complications
5	Severe cerebrovascular accident (for example, ischemic stroke or cerebral hemorrhage) within the pre-analytical within 6 months
6	Glottic edema, allergic rhinitis, respiratory diseases with bronchospasm such as asthma and chronic obstructive lung disease
7	Peripheral vascular disease (for example, Raynaud’s syndrome, intermittent claudication)
8	Patients who needs vasopressor support due to prominent volume retention/overload
9	Moderate-to-severe retinopathy (for example, retinal hemorrhage, visual disturbance, retinal microaneurysm within 6 months)
10	Impaired renal function (serum creatinine ≥ 2.5 mg/dL) or hepatic function (AST or ALT ≥ 3 × ULM)
11	Patients in a clinical status that can significantly influence absorption, distribution, metabolism, and secretion of drugs for clinical trials: History of major gastrointestinal surgery, such as gastrectomy or gastric bypass surgery Inflammatory bowel disease within 12 months Current gastric ulcer, pancreatic function abnormality including pancreatitis, gastrointestinal/rectal bleeding which requires treatment Current urologic stenosis or obstruction which requires treatment
12	Confirmed or suspected drug/alcohol abuse within 6 months
13	Pregnant or lactating women, suspected pregnant women or lactating women
14	Chronic inflammatory diseases which require anti-inflammatory treatment
15	Hypersensitivity to carvedilol
16	Malignant disease including lymphoma and leukemia within 5 years
17	Patients who were prescribed other medication for any other clinical trials within the pre-analytical 28 days
18	Patients who are expected to have prolonged hospital stay due to other medical problems other than chronic heart failure (for example, femoral neck fracture)
19	Patients who are considered inappropriate by researchers to participate in the clinical trial

*ALT* alanine transaminase, *AST* aspartate transaminase, *NT-proBNP* N-terminal pro B-type natriuretic peptide, *ULM* upper limit of meta-stability

### Endpoints

The primary endpoint is the change in N-terminal pro B-natriuretic peptide (NT-proBNP) level from baseline to 6 months after randomization. The secondary endpoints include the proportion of patients with NT-proBNP increment > 10% from baseline, composite of all-cause mortality and readmission, mortality rate, readmission rate, change in systolic and diastolic blood pressure at sitting position, control rate and response rate of blood pressure among patients with elevated blood pressure at baseline, quality of life assessed by the Minnesota Living with Heart Failure Questionnaire and Visual Analog Scale, and drug compliance. Table 2 demonstrates the detailed endpoints.

**Table 2:** Primary and secondary endpoints

	Endpoint details
Primary endpoint	Change in of NT-proBNP level from baseline to 6 months after randomization
Secondary endpoints	The frequency of NT-proBNP increment > 10% from baseline
	Composite of all-cause mortality and readmission
	All-cause mortality rate and readmission rate
	Change in systolic/diastolic blood pressure at sitting position, control/response rate of blood pressure
	Quality of life assessment by the MLHFQ, Visual Analog Scale
	Drug compliance

*MLHFQ* Minnesota Living with Heart Failure Questionnaire, *NT-proBNP* N-terminal pro B-type natriuretic peptide

### Randomization

Subjects who meet the inclusion and exclusion criteria will be randomly assigned to the carvedilol-SR or carvedilol-IR groups, respectively. The randomization table will be generated by an independent statistician using the SAS system’s randomization program. Restricted block randomization is applied in order to ensure that the subjects are assigned to each group in a balanced manner. At this time, the size of the block will be set by a multiple of 2 (for example, 2, 4, 68, etc.) and it will be implemented using the uniform random-number generator in SAS version 9.2. Web-based randomization will be used and all investigators will be masked to this process until the allocation has been determined.

### Carvedilol treatment protocol

In this study, we will use carvedilol (trade name: Dilatrend®) which is manufactured and distributed by Chong Kun Dang Pharmaceutical Corporation in the Republic of Korea. After randomization, patients will receive either carvedilol-IR twice a day or carvedilol-SR once a day for 6 months. Patients in the carvedilol-IR group will receive carvedilol-IR 3.125-mg, 6.25-mg, 12.5-mg, and 25-mg tablets twice a day, while patients in the carvedilol-SR group will receive carvedilol-SR 8-mg, 16-mg, 32-mg, and 64-mg tablets once a day. If the patients were taking beta-blockers other than carvedilol, their dose will be calculated into the equivalent dose of carvedilol (Table 3). The dose of carvedilol can be up-titrated at the discretion of the treating physician.

**Table 3:** Equivalent dose of carvedilol with other beta-blockers

	Dose			
Carvedilol IR	3.125 mg bid	6.25 mg bid	12.5 mg bid	25 mg bid
Carvedilol SR	8 mg qd	16 mg bid	32 mg bid	64 mg qd
Bisoprolol	1.25 mg qd	2.5 mg qd	5 mg qd	10 mg qd
Nebivolol	1.25 mg qd	2.5 mg qd	5 mg qd	10 mg qd
Metoprolol succinate	25 mg qd	50 mg qd	100 mg qd	200 mg qd

*bid* twice daily, *IR* immediate-release, *qd* four times daily, *SR* slow-release

### Prohibited drugs during the clinical trial

To avoid drug-drug interactions that may exert confounding effects, drugs with pharmacodynamic and pharmacokinetic interference will be prohibited during the clinical trial (Table 4).

**Table 4:** Contraindicated drugs during clinical trial

	Contraindication details
1	Other beta-blockers except the study medication
2	Steroid medication (ACTH or corticosteroid). Locally applied drugs are allowed.
3	Non-steroidal anti-inflammatory drugs. They are allowed only for shor term use (≤7 days) according to the examiner’s decision, while their usage is absolutely prohibited 3 days before the scheduled visits. Low-dose aspirin (30 to 300 mg daily) for cardiovascular diseases is allowed.
4	Estrogen medications. If necessary, low-dose hormonal therapy can be continued for therapeutic purpose at the same dose during the study period
5	Sympathomimetic drugs such as catecholamines
6	Anti-psychotics: MAO inhibitors, anti-depressants (tri- and tetra-cyclic classes, selective serotonin reuptake inhibitors including lithium) Sedatives and anti-anxiolytics (benzodiazepines and their antagonists, barbiturates, hypnotics such as zolpidem) can be intermittently used according to the physician's decision, while their usage is absolutely prohibited 7 days before the scheduled visits
7	Immunosuppressants
8	Thyroid hormones. If necessary, thyroid hormonal therapy can be prescribed for therapeutic purpose at the same dose during the study period

*ACTH* adrenocorticotropic hormone, *MAO* monoamine oxidase

### Clinical follow-up

All patients will be clinically followed up at 2, 4, and 6 months after randomization at each participating center. Each clinic visit comprises history taking, physical examination, laboratory tests, and a 12-lead electrocardiograph (ECG). Drug compliance, adverse effects, and additional concomitant drugs will also be checked. Any unscheduled visit will be recorded. The detailed process is described in Table 5.

**Table 5:** Trial process chart

Visit	Visit 1	Visit 2	Visit 3	Visit 4	Visit 5
Status Period	Screening − 2 to 0 week	Baseline 0 months	2 months (± 4 days)	4 months (± 14 days)	6 months (± 14 days)
Inclusion/exclusion criteria	•				
Informed consent	•				
Demographic data	•				
Past medical history^a^	•				
Randomization		•			
Physical examination	•	•	•	•	•
Vital signs	•	•	•	•	•
Height and weight^b^	•	•	•	•	•
Blood analysis^c^	•	•			•
Urinalysis^d^	•	•			•
Pregnancy test^e^	•	•			•
Chest x-ray^f^	•				
12-lead electrocardiograph^g^	•				•
Echocardiography^h^	•	•			•
Concomitant medication	•	•	•	•	•
Adverse events		•	•	•	•
Patient’s compliance			•	•	•
Questionnaire for quality of life		•			•

^a^The history of diseases, medications, and operations will be recorded

^b^Check weight only after visit 2

^c^CBC: WBC with differential count, hemoglobin, hematocrit, platelets; chemistry: albumin, total protein, triglycerides, high-density lipoprotein-cholesterol, low-density lipoprotein-cholesterol, blood urea nitrogen, serum creatinine, uric acid, total bilirubin, AST, ALT, ALP, NT-proBNP, hs-CRP; electrolytes: Na, K, Ca, Cl, P

^d^Specific gravity, pH, albumin (protein), glucose, blood (occult blood), urobilinogen, ketones

^e^Women of child-bearing age

^f,g^Examination at visit 6 can be optionally performed according to the examiner’s decision

^h^Follow-up echocardiograph, if available

### Evaluation of drug compliance

Drug compliance will be assessed by “pill count,” by comparing the number of doses remaining with the number of doses that should remain. All patients will be educated to bring the remaining tablets of carvedilol-IR or -SR to each scheduled visits during the clinical follow-up period. Examiners will count the number of returned pills and calculate the drug compliance as follows:

$$ Drug\kern0.5em \mathrm{compliance}\kern0.5em =\kern0.5em \frac{number\kern0.5em of\kern0.5em pills\kern0.5em dispensed- number\kern0.5em of\kern0.5em pills\kern0.5em returned}{number\kern0.5em of\kern0.5em pills\kern0.5em dispensed} $$

If any discrepancy is present, examiners should record the reason for the difference. Overall medication compliance should be at least 80% or more during the trial. Subjects who do not satisfy this criterion will be excluded from the per-protocol (PP) analysis.

### Statistical analysis

#### Sample size calculation

**Fig. 2:**
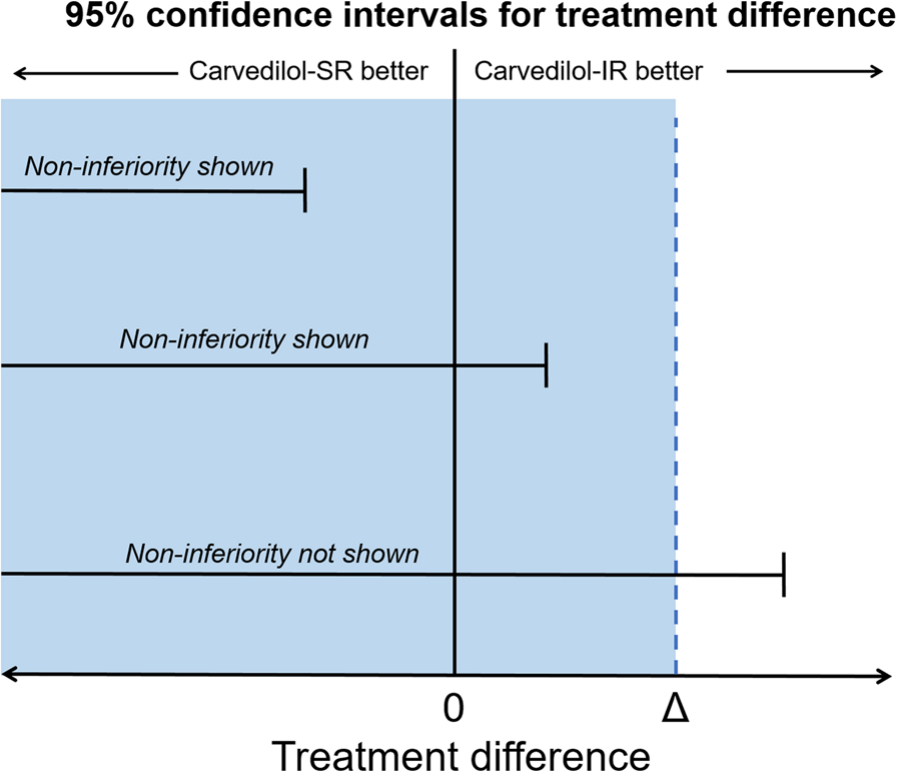
Possible outcomes of the study. Δ stands for the non-inferiority margin

The aim of the study is to evaluate whether the clinical efficacy of carvedilol-SR is non-inferior to carvedilol-IR in terms of NT-proBNP reduction in stable Asian HFrEF patients. Therefore, the sample size was calculated based on the primary endpoint, i.e., the change in NT-proBNP level from baseline to 6 months. Olsson et al. [18] showed a decrease in NT-proBNP level by 334.5 ± 311.3 pg/ml in patients with chronic HF and receiving carvedilol. We established a non-inferiority margin of 110 pg/ml, which corresponds to 33.3% of the mean change. With an estimated dropout rate of 10%, a total of 320 patients (160 patients in each group) would be necessary to provide a power of 85% with a one-sided alpha of 2.5%. This will provide 95% power to demonstrate the non-inferiority of carvedilol-SR once a day compared to carvedilol-IR twice a day (Fig. 2).

#### Statistical analysis

Data will be presented as numbers and frequencies for categorical variables, and as means ± standard deviations or median with interquartile range for continuous variables. For comparisons between the carvedilol-IR group and carvedilol-SR group, the *χ*^2^ test or Fisher’s exact test will be used for categorical variables, and the unpaired Student *t* test will be used for continuous variables, as appropriate. Fisher’s exact test will be used when the expected frequencies are less than 5. The change in NT-proBNP level from baseline to 6 months after randomization will be analyzed with the paired *t* test. In addition, analysis of covariance (ANCOVA) will be also conducted to analyze the change of NT-proBNP. The outcomes of chronological trend, such as mortality and readmission rate, will be assessed using Kaplan-Meier estimates. The log-rank test will be used to compare differences in clinical outcomes between the groups. A multivariable Cox proportional hazard regression model will be used to adjust for significant covariates. The data will be primarily analyzed according to the intention-to-treat rule including all randomized subjects. We also plan a PP analysis for patients with drug compliance of 80% or less. One-sided *p* values < 0.05 will be considered statistically significant. The analyses will be performed by a professional statistician.

### Trial organization

#### Executive Committee

The Executive Committee will be composed of the study chairperson and the principal investigators of the investigating centers. This committee will approve the final trial design and protocol issued to the Data and Safety Monitoring Board (DSMB) and the clinical sites. This committee will also be responsible for reviewing the final results, determining the methods of presentation and publication, and the selection of secondary projects and publications by members of the Steering Committee.

#### Data Safety Monitoring Board (DSMB)

An independent DSMB will be composed of cardiologists and a biostatistician, all of whom did not participate in the trial, following the applicable regulatory guidelines. The DSMB will review the safety data from this study and will make recommendations based on safety analyses of unanticipated device effects (UADEs), serious adverse events (SAEs), protocol deviations, and follow-up reports. In addition to the scheduled DSMB meetings (which will be determined prior to the initiation of the study) the board will convene a meeting at any time if safety problems become an issue. The DSMB is responsible for recommending to the Executive Committee to modify or stop the study if there are any safety or compliance issues. However, the final decision regarding study modifications will rest with the Executive Committee. Cumulative safety data will be reported to the DSMB and will be reviewed on an ongoing basis throughout enrollment and follow-up to ensure patient safety. Every effort will be made to allow the DSMB to conduct an unbiased review of the patients’ safety information. All DSMB reports will be made available to the appropriate agencies upon request but otherwise will remain strictly confidential. Prior to the DSMB’s first review of the data, the DSMB charter will be drafted. This plan defines the stopping rules for stopping the trial for safety. The first meeting of the DSMB will be requested for discussion of the protocol and an understanding of all the protocol elements. The DSMB will develop a consensus understanding of all trial endpoints and definitions used in the event adjudication process.

#### Clinical Event Adjudication Committee (CEAC)

The Clinical Events Adjudication Committee (CEAC) consists of cardiologists who do not participate in this study. The CEAC has responsibility to develop specific criteria used for the categorization of clinical events and clinical endpoints in the study, which are based on protocol. At the onset of the trial, the CEAC establishes regulations mentioning the minimum amount of data required and the algorithm followed in order to classify a clinical event. All members of the CEAC are blinded to the primary results of the study and meet regularly to review and adjudicate all clinical events in which the required minimum data is available. The CEAC also reviews and rules on all deaths that may occur during the trial.

#### Data coordination and site management

Data coordination and site management services are performed by the Clinical Trials Center at Seoul National University Bundang Hospital.

#### Ethical approval

This clinical trial was approved by the Institutional Review Board or Ethics Committee at each of the 13 participating hospitals; they are: Seoul National University Bundang Hospital, Seoul National University Hospital, Yonsei University Severance Hospital, Wonju Severance Christian Hospital, Samsung Medical Center, Sejong General Hospital, Ajou University Hospital, Boramae Medical Center, Hallym University Medical Center, Korea University Guro Hospital, Korea University Anam Hospital, Seoul Medical Center, and Asan Medical Center. The investigation will conform to the principles outlined in the Declaration of Helsinki. The study protocol has been registered at ClinicalTrials.gov (NCT03209180). All authors are responsible for the study design, data acquisition, data analysis, manuscript writing and editing.

## Discussion

After an initial cardiac injury, there is an increase in neuro-humoral activity, i.e., the sympathetic nervous system and renin-angiotensin-aldosterone system [5]. This response can temporarily increase the myocardial contractility [19]; however, sustained over-activation can lead to the development and progression of HF [20]. Thus, angiotensin-converting enzyme inhibitors/angiotensin-receptor blockers and beta-blockers have been used to attenuate the neuro-humoral over-activation and have been shown to improve the prognosis of patients with HFrEF. Unlike to renin-angiotensin system blockers (RAS blockers), only four beta-blockers, i.e., carvedilol, metoprolol succinate, bisoprolol, and nebivolol, have demonstrated a beneficial effect. For example, bucindolol and propranolol failed to show any clinical benefit, suggesting that the beneficiary effect of beta-blockers is not “a class effect” but a substrate-specific effect [6, 7, 21–23]. Therefore, the current clinical practice guidelines for managing HF patients provide detailed information on the drug name and target dose for each agent [1, 11].

Carvedilol has been extensively evaluated in HFrEF patients with mild-to-severe LV dysfunction [8, 9, 24] and has demonstrated remarkable efficacy in improving clinical outcomes [10]. Unlike bisoprolol and metoprolol succinate, carvedilol has a short elimination half-life, so that it needs to be administrated twice a day [12]. Many HF patients have advanced age and have multiple comorbidities, so that they take multiple drugs [2, 25]. A complex medication regimen causes reduced drug compliance and, consequently, can lead to worse clinical outcomes [14, 16, 26]. In addition, the high drug adherence observed in well-controlled clinical trials may differ from that in real-world clinical practice. To increase the drug compliance of carvedilol-IR, longer-acting carvedilol-SR has been developed.

Carvedilol-SR meets the basic criteria for a controlled release agent: (1) it maintains an effective plasma concentration throughout the day and (2) does not exceed the critical value above which side effects often develop [27]. In addition, regarding the pharmacokinetic profile, carvedilol-SR once daily was similar to that for carvedilol-IR given twice daily [27].

The SLOW-HF study is a randomized, open-label. phase-IV clinical trial that investigates the clinical benefit and safety of carvedilol-SR compared to carvedilol-IR in patients with HFrEF. If carvedilol-SR is not inferior to carvedilol-IR in terms of clinical efficacy and safety, it would be a useful therapeutic option to improve medication complexity and compliance in managing HFrEF patients.

## Study limitations

There are several limitations. First, the primary endpoint of our study is the change in NT-proBNP level from baseline to the study end. Although NT-proBNP is a well-validated marker for risk stratification and used as a surrogate marker for clinical outcomes, clinical endpoints, such as mortality and rehospitalization for worsening HF, would be more definitive. Second, there exists a possibility that patients with more advanced HF may be under-represented in this study because they often have fluid volume retention. However, similar findings have been observed in other clinical trials which investigated the effect of beta-blocker management in HF patients [6, 7, 10, 21]. Finally, this is an open-label study.

## Trial status

The trial is currently in the recruitment phase (recruitment began on 27 October 2016 and is expected to finish on 31 December 2018).

## Additional file

Additional file 1: SPIRIT 2013 Checklist. (DOC 124 kb)

## Abbreviations

CEAC: Clinical Event Adjudication Committee
DSMB: Data and Safety Monitoring Board
HF: Heart failure
HFrEF: Heart failure with reduced ejection fraction
IR: Immediate release
LVEF: Left ventricular ejection fraction
NT-proBNP: N-terminal pro B-natriuretic peptide
PP: Per-protocol
SR: Slow release
