# Correction: Assessment of clinical effect and treatment quality of immediate-release carvedilol-IR versus SLOW release carvedilol-SR in Heart Failure patients (SLOW-HF): study protocol for a randomized controlled trial

**DOI:** 10.1186/s13063-022-06559-4

**Published:** 2022-07-25

**Authors:** Dong-Ju Choi, Chan Soon Park, Jin Joo Park, Hae-Young Lee, Seok-Min Kang, Byung-Su Yoo, Eun-Seok Jeon, Seok Keun Hong, Joon-Han Shin, Myung-A Kim, Dae-Gyun Park, Eung-Ju Kim, Soon-Jun Hong, Seok Yeon Kim, Jae-Joong Kim

**Affiliations:** 1 Department of Internal Medicine, Seoul National University College of Medicine, Seoul National University Bundang Hospital, Seongnam, South Korea; 2 Department of Internal Medicine, Seoul National University College of Medicine, Seoul National University Hospital, Seoul, South Korea; 3 Division of Cardiology, Yonsei University Severance Hospital, Seoul, South Korea; 4 Division of Cardiology, Yonsei University Wonju Severance Christian Hospital, Wonju, South Korea; 5 Department of Internal Medicine, Sungkyunkwan University College of Medicine, Samsung Medical Center, Seoul, South Korea; 6 Division or Cardiology, Sejong General Hospital, Bucheon, Gyeonggi-do South Korea; 7 Division of Cardiology, Ajou University Hospital, Suwon, Gyeonggi-do South Korea; 8 Department of Internal Medicine, Seoul National University College of Medicine, Seoul National University Boramae Medical Center, Seoul, South Korea; 9 Cardiovascular Center, Hallym University Medical Center, Seoul, South Korea; 10 Division of Cardiology, Korea University Guro Hospital, Seoul, South Korea; 11 Division of Cardiology, Korea University Anam Hospital, Seoul, South Korea; 12 Department of Internal Medicine, Seoul Medical Center, Seoul, South Korea; 13 Department of Internal Medicine, University of Ulsan College of Medicine, Asan Medical Center, Seoul, South Korea; 14 Cardiovascular Center and Division of Cardiology, Department of Internal Medicine, Seoul National University Bundang Hospital, 82 Gumi-ro 173 Beon-gil, Bundang-gu, Gyeonggi-do 13620 South Korea

**Correction: Trials 19, 103 (2018)**

**https://doi.org/10.1186/s13063-018-2470-5**

Following publication of the original article [1] it was reported that there is an error in the footnotes for Table 3. The incorrect footnote is the following: “*qd* four times daily”. This should be “*qd* once daily”.
