# Supplementary material for: Assessment of clinical effect and treatment quality of immediate-release carvedilol-IR versus SLOW release carvedilol-SR in Heart Failure patients (SLOW-HF): study protocol for a randomized controlled trial
Source: Trials. 2018 Feb 13;19:103. doi: 10.1186/s13063-018-2470-5 (PMC5809818; doi:10.1186/s13063-018-2470-5)
Supplement: Supplementary file 1 — SPIRIT 2013 Checklist. (DOC 124 kb) [file 13063_2018_2470_MOESM1_ESM.doc]

**Additional file 1**.SPIRIT 2013 Checklist


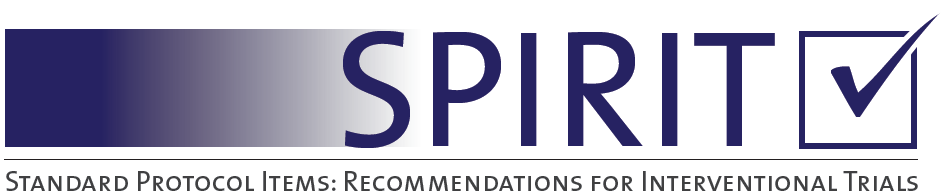


SPIRIT 2013 Checklist: Recommended items to address in a clinical trial protocol and related documents*

| Section/item | ItemNo | Description |
| --- | --- | --- |
| **Administrative information** | | |
| Title | 1  v | Descriptive title identifying the study design, population, interventions, and, if applicable, trial acronym  Assessment of Clinical Effect and Treatment Quality of Immediate Release Carvedilol-IR Versus SLOW Release Carvedilol-SR in HEART FAILURE Patient (SLOW-HF): Study protocol for a randomized controlled trial |
| Trial registration | 2a  v | Trial identifier and registry name. If not yet registered, name of intended registry  <https://clinicaltrials.gov/ct2/show/NCT03209180>  ClinicalTrials.gov: NCT03209180, Registered on 6 July 2017 |
|  | 2b | All items from the World Health Organization Trial Registration Data Set N/A |
| Protocol version | 3  v | Date and version identifier  SLOW-HF Version 4.2 / Effective date 9 March 2017 |
| Funding | 4  v | Sources and types of financial, material, and other support  This project is an investigator-initiated trial and is funded by an unrestricted grant from Chong Kun Dang Pharmaceutical Corporation. |
| Roles and responsibilities | 5a  v | Names, affiliations, and roles of protocol contributors  JJP: Study design, Data collection, Manuscript writing, Patient recruitment, Corresponding author  D-JC, CSP: Study design, Manuscript writing, Data analysis  H-YL, S-MK, B-SY, E-SK, SKH, J-HS, M-AK, D-GP, E-JK, S-JH, SYK: patient recruitment, J-JK, Data analysis, Patient recruitment |
|  | 5b  v | Name and contact information for the trial sponsor  Chong Kun Dang Pharmaceutical Corporation  [www.ckdpharm.com](http://www.ckdpharm.com/)  +82-2-2194-0300 |
|  | 5c  v | Role of study sponsor and funders, if any, in study design; collection, management, analysis, and interpretation of data; writing of the report; and the decision to submit the report for publication, including whether they will have ultimate authority over any of these activities  Chong Kun Dang Pharmaceutical Corporation, the funding source has no involvement in the current study. |
|  | 5d  v | Composition, roles, and responsibilities of the coordinating centre, steering committee, endpoint adjudication committee, data management team, and other individuals or groups overseeing the trial, if applicable (see Item 21a for data monitoring committee)  Explained in Paper |
| Introduction |  |  |
| Background and rationale | 6a  v | Description of research question and justification for undertaking the trial, including summary of relevant studies (published and unpublished) examining benefits and harms for each intervention  Explained in Paper |
|  | 6b  v | Explanation for choice of comparators  Explained in Paper |
| Objectives | 7  v | Specific objectives or hypotheses  Explained in Paper |
| Trial design | 8  v | Description of trial design including type of trial (eg, parallel group, crossover, factorial, single group), allocation ratio, and framework (eg, superiority, equivalence, noninferiority, exploratory)  Explained in Paper |
| Methods: Participants, interventions, and outcomes | | |
| Study setting | 9  v | Description of study settings (eg, community clinic, academic hospital) and list of countries where data will be collected. Reference to where list of study sites can be obtained  Explained in Paper |
| Eligibility criteria | 10  v | Inclusion and exclusion criteria for participants. If applicable, eligibility criteria for study centres and individuals who will perform the interventions (eg, surgeons, psychotherapists)  Explained in Paper |
| Interventions | 11a  v | Interventions for each group with sufficient detail to allow replication, including how and when they will be administered  Explained in Paper |
|  | 11b  v | Criteria for discontinuing or modifying allocated interventions for a given trial participant (eg, drug dose change in response to harms, participant request, or improving/worsening disease)  Explained in Paper |
|  | 11c  v | Strategies to improve adherence to intervention protocols, and any procedures for monitoring adherence (eg, drug tablet return, laboratory tests)  Explained in Paper |
|  | 11d  v | Relevant concomitant care and interventions that are permitted or prohibited during the trial  Explained in Paper |
| Outcomes | 12  v | Primary, secondary, and other outcomes, including the specific measurement variable (eg, systolic blood pressure), analysis metric (eg, change from baseline, final value, time to event), method of aggregation (eg, median, proportion), and time point for each outcome. Explanation of the clinical relevance of chosen efficacy and harm outcomes is strongly recommended  Explained in Paper |
| Participant timeline | 13  v | Time schedule of enrolment, interventions (including any run-ins and washouts), assessments, and visits for participants. A schematic diagram is highly recommended (see Figure)  Explained in Paper |
| Sample size | 14  v | Estimated number of participants needed to achieve study objectives and how it was determined, including clinical and statistical assumptions supporting any sample size calculations  Explained in Paper |
| Recruitment | 15  v | Strategies for achieving adequate participant enrolment to reach target sample size  Explained in Paper |
| **Methods: Assignment of interventions (for controlled trials)** | | |
| Allocation: |  |  |
| Sequence generation | 16a  v | Method of generating the allocation sequence (eg, computer-generated random numbers), and list of any factors for stratification. To reduce predictability of a random sequence, details of any planned restriction (eg, blocking) should be provided in a separate document that is unavailable to those who enrol participants or assign interventions  Explained in Paper |
| Allocation concealment mechanism | 16b  v | Mechanism of implementing the allocation sequence (eg, central telephone; sequentially numbered, opaque, sealed envelopes), describing any steps to conceal the sequence until interventions are assigned  Explained in Paper |
| Implementation | 16c  v | Who will generate the allocation sequence, who will enrol participants, and who will assign participants to interventions  Explained in Paper |
| Blinding (masking) | 17a  v | Who will be blinded after assignment to interventions (eg, trial participants, care providers, outcome assessors, data analysts), and how  Explained in Paper |
|  | 17b  v | If blinded, circumstances under which unblinding is permissible, and procedure for revealing a participant’s allocated intervention during the trial  This is an open label study and blinding was not done |
| **Methods: Data collection, management, and analysis** | | |
| Data collection methods | 18a  v | Plans for assessment and collection of outcome, baseline, and other trial data, including any related processes to promote data quality (eg, duplicate measurements, training of assessors) and a description of study instruments (eg, questionnaires, laboratory tests) along with their reliability and validity, if known. Reference to where data collection forms can be found, if not in the protocol  Explained in Paper |
|  | 18b  V | Plans to promote participant retention and complete follow-up, including list of any outcome data to be collected for participants who discontinue or deviate from intervention protocols  Explained in Paper |
| Data management | 19  v | Plans for data entry, coding, security, and storage, including any related processes to promote data quality (eg, double data entry; range checks for data values). Reference to where details of data management procedures can be found, if not in the protocol  Explained in Paper |
| Statistical methods | 20a  v | Statistical methods for analysing primary and secondary outcomes. Reference to where other details of the statistical analysis plan can be found, if not in the protocol  Explained in Paper |
|  | 20b  v | Methods for any additional analyses (eg, subgroup and adjusted analyses)  Explained in Paper |
|  | 20c  v | Definition of analysis population relating to protocol non-adherence (eg, as randomised analysis), and any statistical methods to handle missing data (eg, multiple imputation)  Explained in Paper |
| **Methods: Monitoring** | | |
| Data monitoring | 21a  v | Composition of data monitoring committee (DMC); summary of its role and reporting structure; statement of whether it is independent from the sponsor and competing interests; and reference to where further details about its charter can be found, if not in the protocol. Alternatively, an explanation of why a DMC is not needed  Explained in Paper |
|  | 21b  v | Description of any interim analyses and stopping guidelines, including who will have access to these interim results and make the final decision to terminate the trial  Explained in Paper |
| Harms | 22  v | Plans for collecting, assessing, reporting, and managing solicited and spontaneously reported adverse events and other unintended effects of trial interventions or trial conduct  Explained in Paper |
| Auditing | 23  v | Frequency and procedures for auditing trial conduct, if any, and whether the process will be independent from investigators and the sponsor  Explained in Paper. The funding source has no involvement in the current study. |
| Ethics and dissemination | | |
| Research ethics approval | 24  v | Plans for seeking research ethics committee/institutional review board (REC/IRB) approval  Explained in Paper |
| Protocol amendments | 25  v | Plans for communicating important protocol modifications (eg, changes to eligibility criteria, outcomes, analyses) to relevant parties (eg, investigators, REC/IRBs, trial participants, trial registries, journals, regulators)  N/A |
| Consent or assent | 26a  v | Who will obtain informed consent or assent from potential trial participants or authorised surrogates, and how (see Item 32)  Explained in Paper |
|  | 26b  v | Additional consent provisions for collection and use of participant data and biological specimens in ancillary studies, if applicable  N/A |
| Confidentiality | 27  v | How personal information about potential and enrolled participants will be collected, shared, and maintained in order to protect confidentiality before, during, and after the trial  Explained in Paper |
| Declaration of interests | 28  v | Financial and other competing interests for principal investigators for the overall trial and each study site  None declared |
| Access to data | 29  v | Statement of who will have access to the final trial dataset, and disclosure of contractual agreements that limit such access for investigators  Explained in Paper |
| Ancillary and post-trial care | 30  v | Provisions, if any, for ancillary and post-trial care, and for compensation to those who suffer harm from trial participation  Explained in Paper |
| Dissemination policy | 31a  v | Plans for investigators and sponsor to communicate trial results to participants, healthcare professionals, the public, and other relevant groups (eg, via publication, reporting in results databases, or other data sharing arrangements), including any publication restrictions  Explained in Paper |
|  | 31b  v | Authorship eligibility guidelines and any intended use of professional writers  Explained in Paper |
|  | 31c  v | Plans, if any, for granting public access to the full protocol, participant-level dataset, and statistical code  N/A |
| Appendices |  |  |
| Informed consent materials | 32  v | Model consent form and other related documentation given to participants and authorised surrogates  Explained in Paper |
| Biological specimens | 33  v | Plans for collection, laboratory evaluation, and storage of biological specimens for genetic or molecular analysis in the current trial and for future use in ancillary studies, if applicable N/A |

*It is strongly recommended that this checklist be read in conjunction with the SPIRIT 2013 Explanation & Elaboration for important clarification on the items. Amendments to the protocol should be tracked and dated. The SPIRIT checklist is copyrighted by the SPIRIT Group under the Creative Commons “[Attribution-NonCommercial-NoDerivs 3.0 Unported](http://www.creativecommons.org/licenses/by-nc-nd/3.0/)” license.
